# An *in silico *analysis of the mitochondrial protein import apparatus of plants

**DOI:** 10.1186/1471-2229-10-249

**Published:** 2010-11-16

**Authors:** Chris Carrie, Monika W Murcha, James Whelan

**Affiliations:** 1 Australian Research Council Centre of Excellence in Plant Energy Biology, University of Western Australia, 35 Stirling, Crawley 6009, WA, Australia

## Abstract

**Background:**

An *in silico *analysis of the mitochondrial protein import apparatus from a variety of species; including *Chlamydomonas reinhardtii*, *Chlorella variabilis, Ectocarpus siliculosus*, *Cyanidioschyzon merolae*, *Physcomitrella patens*, *Selaginella moellendorffii, Picea glauca*, *Oryza sativa *and *Arabidopsis thaliana *was undertaken to determine if components differed within and between plant and non-plant species.

**Results:**

The channel forming subunits of the outer membrane components Tom40 and Sam50 are conserved between plant groups and other eukaryotes. In contrast, the receptor component(s) in green plants, particularly Tom20, (*C. reinhardtii, C. variabilis, P. patens, S. moellendorffii, P. glauca, O. sativa and A. thaliana*) are specific to this lineage. Red algae contain a Tom22 receptor that is orthologous to yeast Tom22. Furthermore, plant mitochondrial receptors display differences between various plant lineages. These are evidenced by distinctive motifs in all plant Metaxins, which are absent in red algae, and the presence of the outer membrane receptor OM64 in Angiosperms (rice and Arabidopsis), but not in lycophytes (*S. moellendorffii*) and gymnosperms (*P. glauca*). Furthermore, although the intermembrane space receptor Mia40 is conserved across a wide phylogenetic range, its function differs between lineages. In all plant lineages, Tim17 contains a C-terminal extension, which may act as a receptor component for the import of nucleic acids into plant mitochondria.

**Conclusions:**

It is proposed that the observed functional divergences are due to the selective pressure to sort proteins between mitochondria and chloroplasts, resulting in differences in protein receptor components between plant groups and other organisms. Additionally, diversity of receptor components is observed within the plant kingdom. Even when receptor components are orthologous across plant and non-plant species, it appears that the functions of these have expanded or diverged in a lineage specific manner.

## Background

The endosymbiotic event giving rise to the origin of mitochondria is thought to have occurred 1 to 2 billion years ago [1,2]. Details of the conditions that favoured this event and the exact identity of the host cell that engulfed the α-proteobacterial cell are still unclear. It has been proposed that the endosymbiosis that gave rise to mitochondria occurred under anaerobic conditions, followed by early diversification of eukaryotic cells [3]. For plastids, an endosymbiotic event occurred ~1 billion years ago when a heterocyst forming cyanobacterium was engulfed [4,5]. Over time the loss and/or transfer of genes and genomes from the endosymbionts to the host cell nucleus has resulted in the formation of organelles with limited coding capacity [6-8]. The majority of proteins located in mitochondria and plastids are encoded by nuclear located genes, translated in the cytosol and imported into these organelles. Notably, the proteomes of both mitochondria and chloroplasts are derived from a variety of sources and are not simply a subset of the proteins derived from the ancestral endosymbiont [9]. In the most extreme cases, it is thought that all genes that were present in the endosymbiont have been lost, resulting in specialized organelles such as hydrogenosomes and mitosomes [10].

Although mitochondria have a single origin there is variation observed between different mitochondria present in the major branches of life [10]. Mitochondria in plants contain many unique features compared to their fungal or animal counterparts. These include a larger genome, ranging from 200 Kb to 2000 Kb in size [11], extensive *cis *and *trans *splicing of introns, [12], relatively slow rates of mutations [13,14], extensive editing of mRNA [15] and incorporation of foreign DNA [16]. Another notable feature is the presence of a branched respiratory chain [17]. Although fungi contain alternative NAD(P)H dehydrogenases and an alternative oxidase, these are usually only expressed under conditions where the cytochrome chain is inhibited [17]. In contrast, plant mitochondria contain components of the alternative respiratory pathways which exhibit both constitutive and stress induced expression [18]. Furthermore, mitochondria of plants and animals have diversified in a lineage specific manner to include or exclude various biochemical pathways, such as the β-oxidation of fatty acids that occurs in peroxisomes in plants and mitochondria in animals [19].

In plants, the presence of plastids in cells also adds to the complexity of protein sorting required to avoid mis-targeting of proteins to organelles. Plastidic and mitochondrial targeting signals, referred to as transit peptides and presequences respectively, are typically located at the N-terminal end of the protein and are enriched in positively charged residues such as lysine and arginine [20]. It is not known how mis-sorting of proteins is prevented between plastids and mitochondria. A combination of the predicted ability of transit peptides and presequences to form different secondary structures, the proposed presence of cytosolic targeting factors and even targeting of mRNA to the surface of organelles, may all combine to achieve the observed specificity of protein targeting [21,22]. There is a mechanistic difference between recognition of targeting signals by preprotein receptor proteins in plastids and mitochondria, the former involving a GTP/GDP cycle while no energy requirement is observed for receptor binding in mitochondria. This mechanism among others may contribute to the specificity of targeting signal recognition at the surface of each organelle [23,24].

Our knowledge of the mitochondrial protein import apparatus in plants, both experimental and predicted, is largely derived from studies in Arabidopsis, and to a lesser extent from *Solanum tuberosum *(potato). Purification of the translocase of the outer membrane (TOM) complex from both Arabidopsis and potato revealed that Tom40 and Tom7 are orthologous with those from yeast, while Tom20 is not orthologous to yeast or mammalian proteins [25-27]. The other import receptors characterized in yeast (and mammals), Tom70 and Tom22, appear to be absent [28,29]. It has been shown that plant Tom9 is the most likely equivalent to yeast Tom22, but lacks the cytosolic receptor domain [30]. The mitochondrial processing peptidase has been purified from potato and shown to be integrated into the cytochrome bc_1 _complex [31,32]. This is also the case in lower plants examined both in the elkhorn fern *Platycerium bifurcatum *and the field horsetail *Equisetum arvense *[33]. Biochemical purification of the presequence degradation peptidase (PreP) has shown that it is a dual targeted protein and that it is a zinc metalloprotease [34]. Biochemical studies have shown that small intermembrane space proteins also mediate mitochondrial carrier protein import in potato mitochondria. In addition, the plant TIM17:23 complex differs to that in yeast in that the Tim17 in Arabidopsis contains a C-terminal extension that must be removed before it can complement a *tim17 *mutant in yeast [35,36].

**Figure 1:**
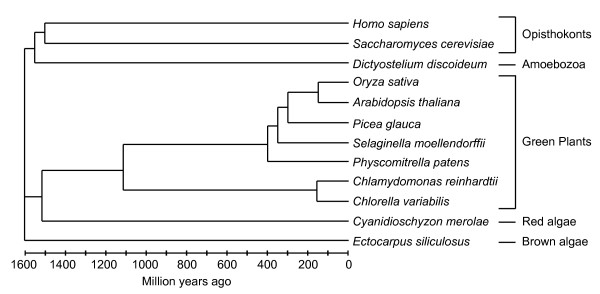
**Overview of the evolutionary relationship of organisms used in this study**. The taxonomy database at NCBI was used to draw a phylogenetic tree, which was visualized using PHY-PI [91]. The timeline is based upon [57].

However, there are limited studies on the nature of the mitochondrial protein import apparatus from other plants, ranging from single celled algae to monocots. Thus, in order to gain a better overview of the protein import apparatus in plants, compared to fungal and animal counterparts, an *in silico *analysis of these components was carried out. This was based on the fact that complete genome sequences now exist for the single celled green algae, *Chlamydomonas reinhardtii *(Chlorophyte) [37] and *Chlorella variabilis*, an intracellular single celled green algae photosynthetic symbiont in *Paramecium bursaria *[38], a moss, *Physcomitrella patens *(Bryophyte) [39], *Selaginella moellendorffii*, an ancient vascular plant [40], and higher plants *Oryza sativa *[41], *Arabidopsis thaliana *[42] and *Picea glauca *[43] (Spermatophytes) (Figure 1). We have also included analysis from brown algae, *Ectocarpus siliculosus *(Phaeophyceae) [44] and the red algae, *Cyanidioschyzon merolae *(Rhodophyta) [45] (Figure 1). Red algae represent a cell lineage with a primary plastid endosymbiosis that is proposed to have been derived from the same event that gave rise to the plastids in green plants, but diverged from the green plant lineage early after this endosymbiotic event [46]. Brown algae have obtained their plastids via a secondary endosymbiosis, and contain four plastid envelope membranes. Thus, plastid proteins are first targeted to the outer membrane via a hydrophobic signal sequence and secondary targeting signals mediate uptake into plastids [47].

## Results and Discussion

### Translocase of the Outer Membrane (TOM)

The TOM complex represents the gateway into mitochondria, through which almost all mitochondrial proteins pass (exceptions include Fis1 [48]). It has been characterized from yeast, Neurospora, mammals and plants, in particular Arabidopsis. In addition to being purified from Arabidopsis, functional studies on the Tom20 receptor components show that all three isoforms can be deleted, resulting in a reduced rate of import for several precursor proteins, but no deleterious phenotypic lesions. The complex typically contains 7 subunits, Tom70, 40, 22, 20 7, 6, and 5, but in Arabidopsis Tom22 is replaced by Tom9 and no orthologue to Tom70 can be identified. Arabidopsis contain a protein termed OM64 that is not present in yeast or mammals, which appears to play a role as an import receptor.

**Figure 2:**
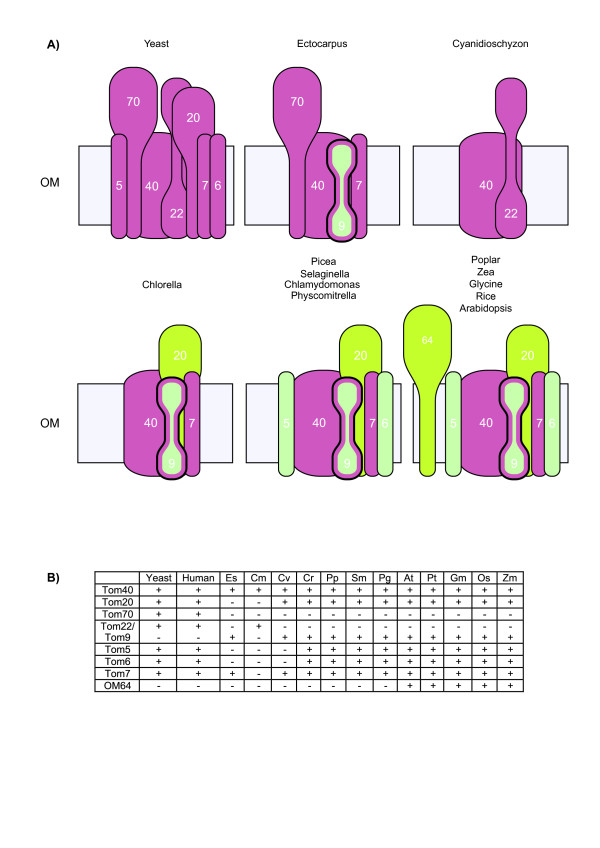
**Diversity of the TOM complex in plants**. A) Schematic diagrams of the TOM complex from a selection of plant species using the TOM complex from yeast as a reference. B) A table depicting components of the TOM complex in a variety of organisms. The pink color refers to proteins that are conserved across all organisms and likely have a common ancestor. The lime green colored proteins are specific to the plant lineage. The pale green proteins are proteins that have an unknown origin. Yeast - *Saccharomyces cerevisiae*, Ectocarpus - *Ectocarpus siliculosus *(Es), Cyanidioschyzon - *Cyanidioschyzon merolae *(Cm), Chlorella - *Chlorella variabilis *(Cv), Picea - *Picea glauca *(Pg), Selaginella - *Selaginella moellendorffii *(Sm), Chlamydomonas - *Chlamydomonas reinhardtii *(Cr), Physcomitrella - *Physcomitrella patens *(Pp), Rice - *Oryza sativa *(Os), Arabidopsis - *Arabidopsis thaliana *(At), Human - *Homo sapiens*, Poplar -*Populus tricocarpa *(Pt), Glycine - *Glycine max *(Gm) Zea - *Zea mays *(Zm).

The TOM complex fulfills the vital function of specifically recognizing mitochondrial proteins from the pool of all proteins synthesized in the cytosol. Of the seven components characterized biochemically to be present in the TOM complex from yeast, only Tom40 is conserved between yeast, mammals and plants (Figure 2, Additional file 1). While Tom40 is a β-barrel protein, there is no significant sequence similarity with bacterial β-barrel proteins [30], nonetheless, hidden Markov model searches define this as a universal component of all mitochondria, including mitosomes in *Entamoeba histolytica *and *Giardia intestinalis *[49,50].

**Figure 3:**
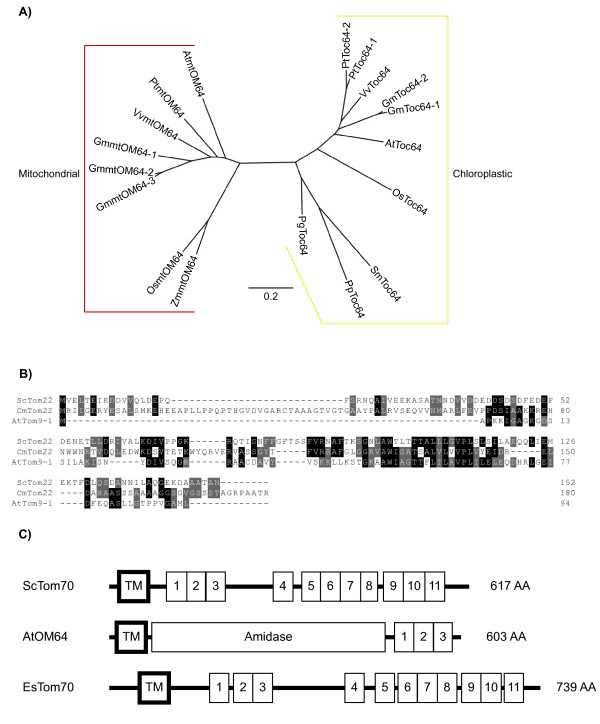
**Analysis of components of the plant TOM complex**. A) Phylogenetic tree of the chloroplastic Toc64 and mitochondrial OM64 sequences from plants. Only sequences which showed the characteristic domain structure of Toc64, an N-terminal transmembrane domain followed by an amidase like domain with 3 TPR repeats at the C-terminus, were used for phylogenetic analyses. No such proteins were identified in *Ectocarpus siliculosus*, *Cyanidioschyzon merolae*, *Chlamydomonas reinhardtii *and *Chlorella variabilis*. For most plant species a clear Toc64 and mtOM64 homologue can be identified however only 1 sequence can be identified in *Physcomitrella patens*, *Selaginella moellendorffii *and *Picea glauca *which all branch closest to the Toc64 chloroplastic proteins. B) Sequence alignment of the *Saccharomyces cerevisiae *Tom22 with the *Arabidopsis thaliana *Tom9 and *Cyanidioschyzon merloae *Tom22. While most plants contain only the Arabidopsis Tom9 like protein Cyanidioscyzon contains a full Tom22 receptor, which shows a high similarity with the yeast Tom22. C) The domain organization of the yeast Tom70, Arabidopsis mtOM64 and an EsTom70 like protein. TM - transmembrane domain and the numbers correspond to the TPR repeats. At - *Arabidopsis thaliana*, Vv - *Vitis vinifera*, Gm - *Glycine max*, Pt - *Populus tricocarpa*, Os - *Oryza sativa*, Pp - *Physcomitrella patens*, Zm - *Zea mays*, Sc - *Saccharomyces cerevisiae*, Cm - *Cyanidioschyzon merolae*, Es - *Ectocarpius siliculosus*, Sm - *Selaginella moellendorffii *and Pg - *Picea glauca*.

Tom22 has been shown to fulfill a central receptor role in yeast, and insertional inactivation in yeast results in a strong impairment of mitochondrial biogenesis, compared to the other two preprotein receptors characterized, Tom20 and Tom70 [51]. Searching the genome of the red algae *C. merolae *for Tom22-like proteins identified a protein with a predicted molecular mass of 20 kDa. This protein displays sequence identity and a similar domain structure to the yeast Tom22 (Figure 2, Figure 3b). Thus, the TOM complex of *C. merolae *appears to be similar to that of *D. discoideum*, in that it contains a single receptor Tom22-like protein [49]. In contrast, green plants and *E. siliculosus *do not contain a Tom22 protein. Rather, they contain a Tom9 protein domain component (Figure 2, Additional file 1). Plant Tom9 is predicted to be structurally similar to yeast and mammalian Tom22, except that it lacks the cytosolic receptor domain [30]. Thus, Tom22 has either lost the receptor domain to form Tom9 or been replaced by a different protein. Irrespective of the mechanisms by which Tom9 arose, it appears that green plants have lost the Tom22 receptor. The presence of the Tom22 receptor component in *C. merolae *and *D. discoideum *suggests that it represented a universal mitochondrial receptor component prior to the divergence event that gave rise to plants verse animals and fungi.

None of the receptor proteins characterized in yeast or mammalian systems, Tom20, Tom70 and Tom22, are present in green plants [52,53] (Figure 2). The evolutionary situation for Tom22 is outlined above, and although a Tom20 receptor protein is present in plants, it represents a case of convergent evolution that has been previously well described [27,54,55], thus, plant and yeast Tom20 proteins are not orthologous (Figure 2, Additional file 1). The third receptor component, Tom70, is only present in animals and fungi [45] (Figure 1). Tom70 is not present in any green plant genome [29], a variety of searches in this study failed to detect any Tom70 like sequences in the green plant genomes interrogated. However, in the genome of the brown algae *E. siliculosus*, a protein with a similar domain structure to Tom70 was identified (Figure 3C). This Tom70 like protein contains an N-terminal transmembrane domain and 11 Tetratricopeptide repeat (TPR) motifs similar to yeast Tom70 [29]. However, the level of sequence identity is low (20%), and it is unclear if this protein represents a Tom70 orthologue.

A protein with a predicted molecular mass of 64 kDa (OM64) is found on the outer membrane of mitochondria in Arabidopsis, displaying ~70% sequence identity with the Toc64 protein (translocase of the outer envelope of chloroplasts) from plastids [56] (Figure 3A). In plant mitochondria, this protein has been shown to be involved in the import of some precursor proteins [53]. Analysis of various plant genomes reveals that OM64 appears to be present only in a sub-set of vascular plants and is absent in *P. glauca *and *S. moellendorffii*, as well as lower plant groups represented by *C. reinhardtii*, *C. variabilis, P. patens, E. siliculosus *and *C. merolae *(Figure 2A). Tom7 represents an interesting case in that it is absent in *C. merolae*, but present in all other plants and eukaryotes (Figure 2). TBlastx and hidden Markov model based searches of all red algae genomes available failed to find this component [50]. Thus even if it was not annotated in the genome sequence of *C. merolae *these searches should detect its presence. However, it cannot be ruled out that it may have been missed in the sequencing and/or assembly of the *C. merolae *genome. Tom5 and Tom6, proteins of approximately 50 amino acids long, were not detected in *C. variabilis*, using either plant or yeast interrogation sequences in searches. However, the small size of Tom5 and 6 proteins means that it is difficult to define their evolutionary relationship across wide phylogenetic gaps. Tom7, on the other hand appears to be orthologous across all groups, with the exception that it cannot be found in *C. merolae*. Thus suggesting that the small TOM proteins may be lineage specific, as is the case of the Tom20 receptor.

It is evident that the TOM complex of plants displays diversity with respect to the receptor components present. While Tom40 is universally present, the presence of Tom20 is only evident in green plants, Tom70 is only present in *E. siliculosus*, and OM64 appears to have arisen by a relatively recent evolutionary event as it is only present in a variety of monocot and dicot plants examined and could not be detected in *P. glauca *and *S. moellendorffii *(Figure 2A and 3A). The brown algae *E. siliculosus*, contains a Tom70 type receptor. As there is no Tom70 like sequences in green plants [29], the Tom70 type receptor was either derived from the specific host in the symbiosis that led to the formation of brown algae, or alternatively, it may represent a case of convergent evolution, as has been observed between green plants and Opisthokonts for the Tom20 receptor [27,55].

An analysis of the mitochondrial protein import apparatus in a variety of plants reveals that *C. merolae *clearly contains a Tom22 type receptor in contrast to all other plant lineages. Thus, this component may either have been lost from brown algae and green plant lineages or the presence of a Tom22 type receptor in *C. merolae *represents another case of convergent evolution. As brown algae are proposed to have been derived from red algae, after the latter branched from green plants [57], the Tom22 receptor would have to be lost independently in green plants and brown algae. However, caution needs to be exercised, as the sequence of *E. siliculosus *may not be fully representative of all brown algae.

The question of how red algae solve the sorting problem between plastids and mitochondria may relate to the binding substrates of the receptors, that is, the targeting signals. Analysis of plastid targeting signals from all plant lineages reveals that red algae (and the other primary plant lineage, glaucophytes) contain a phenylalanine residue within a few amino acids of the N-terminus, which is in a hydrophobic context [58]. This 'ancestral' plastid targeting motif is not present in plastid targeting signals in green plants [58], and thus the differentiation of plastid and mitochondrial targeting signals in green plants differs to red algae. In red algae, the "phenylalanine containing" transit peptide may serve as a means for mitochondria and plastid targeting signals to be recognized or rejected by plastidic or mitochondrial receptors respectively.

### Sorting and Assembly Machinery of the Outer Mitochondrial Membrane (SAM)

**Figure 4:**
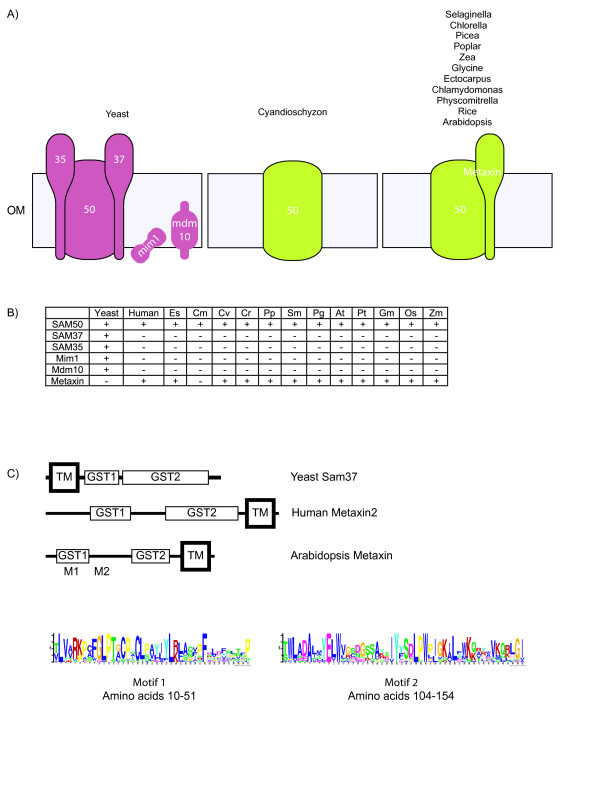
**The SAM complex of plants**. A) Schematic representation of the SAM complexes found in yeast and plants. B) Table indicating the presence or absence of a component of the SAM complexes found in plants. C) Representation of the different domains of yeast Sam37, Human Metaxin and Arabidopsis metaxin proteins. All three share similar Glutathione *S*-transferase (GST) domains. However the location of the transmembrane domains (TM) differs. A motif analysis search of plant metaxin sequences identified Motif 1 to regions between amino acids 37 and 79 and motif 2 between amino acids 104 and 155 in Arabidopsis. These motifs appear only in plant like Metaxins and near a region required for protein binding. Colors and abbreviations are the same as Figure 2.

The SAM complex is required for the insertion of β-barrel and α-helical proteins into the outer membrane [52]. The insertion of β-barrel proteins into the mitochondrial outer membrane is conserved from bacteria to mitochondria and plastids, where Omp85, Sam50 and Toc75 are orthologous β-barrel proteins that are essential for this process [59,60]. However, apart from this central component, there are no other conserved components identified for the insertion of β-barrel proteins into membranes from bacteria to mitochondria and plastids (Figure 4A and 4B). In yeast, four additional components are involved; Sam35, Sam37, Mdm10 and Mim1, with Sam35 representing an essential component. As the SAM complex has not been biochemically characterised from mammalian or plant systems, any additional components are unknown in these systems. The genome of *D. discoideum *has a gene encoding Sam50, but lacks the other components identified in yeast. As *D. discoideum *is an amoeba that diverged from Opisthokonts after this lineage had split from plants, this suggests that the additional components observed in yeast arose after the lineage divergence of plants from other groups. Although additional components are likely to be present in the SAM complexes from plants, they are unlikely to be orthologous to the components in yeast.

In Arabidopsis, a protein called Metaxin has been shown to be involved in the import of β-barrel proteins into the outer membrane. The deletion of Metaxin is not lethal in Arabidopsis, although plants are sterile and grow poorly [53]. Deletion of Metaxin results in a large up-regulation of transcript abundance for genes encoding the mitochondrial β-barrel proteins porin and Tom40, with an accumulation of porin observed in the cytosol, indicating that Metaxin plays a role in the insertion of β-barrel proteins in plants. Mammalian genomes contain two genes encoding Metaxin, in fact the plant Metaxin protein was identified using blast searches of the mammalian Metaxin protein [53] (Figure 4C). Mammalian Metaxin has also been shown to be involved in the import of β-barrel proteins into the outer membrane of mitochondria [61]. Mammalian Metaxin does identify with Sam37 in a blast search, although the sequence identity is very low (Additional file 1). A number of features distinguish plant and animal Metaxins from Sam37 in yeast. Firstly, human and Arabidopsis Metaxins are anchored to the outer membrane in the opposite orientation compared to Sam37 (Figure 4C). Yeast Sam37 is anchored to the mitochondrial outer membrane by an N-terminal transmembrane domain, whereas human and Arabidopsis Metaxins contain C-terminal transmembrane domains. Secondly, human and Arabidopsis Metaxins contain conserved glutathione S-transferase (GST) domains (Figure 4C). Plant and animal Metaxins are distinguished by the fact that Metaxin is not found in a complex with human Sam50 [61], whereas plant Metaxin is in a complex with plant Sam50 (Duncan and Whelan - unpublished data). In Arabidopsis Metaxin there are two conserved motifs in a region critical for binding that are only found in plant type Metaxins (Figure 4C motif 1 and 2). It is also of interest to note that while Trypanosomes do contain a Metaxin like protein [62], there are no Metaxin or Sam37 like proteins identified in *D. discoideum *[49] or *C. merolae *in this study. The presence of a Metaxin protein in *E. siliculosus *may be derived from the host cell. Thus, plant and animal Metaxins may be orthologous, but functions are likely to have diverged over time. Biochemical characterization of the plant SAM complex would provide information on the accessory proteins of this complex and provide a clearer picture of the evolutionary nature of the accessory subunits in this complex.

### Intermembrane space - Mitochondrial intermembrane space import and Assembly (MIA) and Tiny TIMs

**Figure 5:**
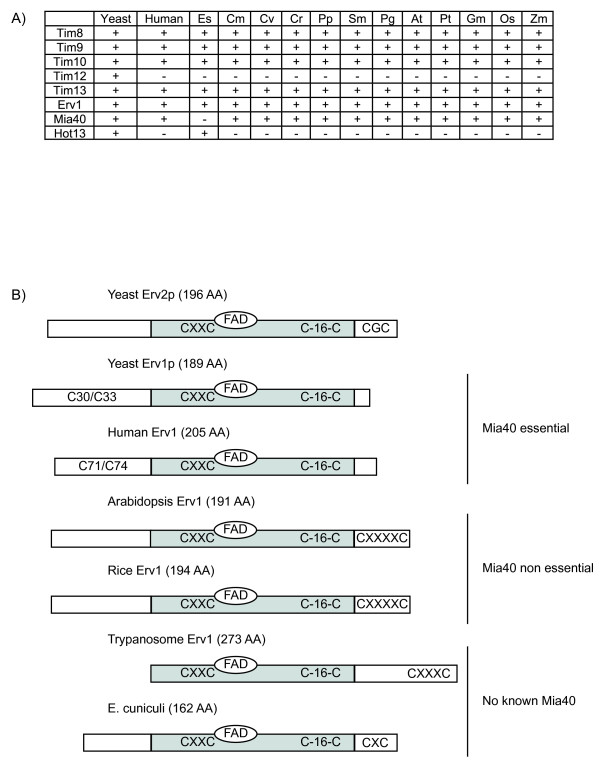
**Components of the MIA and IMS protein import apparatus of plants**. A) A table displaying the components of the small Tim proteins and MIA pathway components of plants. B) Schematic diagram of the different Erv1 sequences found in different organisms. The grey region represents the most conserved region between different Erv1 sequences containing the redox centre, the FAD binding site and two conserved cysteine domains (CXXC and C-16-C). The location of the third cysteine pair differs between organisms which seems to be dependent on either a Mia40 protein being present or whether that Mia40 is essential or not. Abbreviations are the same as Figures 2 and 4.

The intermembrane space contains two sets of proteins that are essential for cell viability in yeast. The tiny TIM proteins 8, 9, 10 and 13 appear to be present in a wide variety of eukaryotes (Figure 5A). They play an essential role in the import of carrier proteins into the inner membrane and also the assembly of β-barrel proteins into the outer membrane [52]. It has been proposed that they arose from an ancestral protein present in the original host that housed the mitochondrial endosymbiont [63]. There are eukaryotes that lack the small Tims (*Trichomonas vaginalis and Encephalitozoon cuniculi) *or only contain one small Tim protein (*Cryptosporidium hominis) *[64,65], indicating that they are not absolutely essential, even though these organisms contain carrier type proteins on the inner membrane that should require these components for import. Thus, the lack of small Tims is likely to be a derived situation associated with the presence of highly modified mitochondria (i.e. mitosomes) in these organisms.

The MIA pathway is the most recently described import pathway for mitochondrial proteins. It consists of two essential proteins in yeast, Mia40 and Erv1, which catalyse the oxidative folding of proteins when they enter the intermembrane space. Substrates of this pathway are proteins that contain conserved cysteine residues that undergo oxidative protein folding in the intermembrane space. Both Mia40 and Erv1 are essential proteins in yeast, with Mia40 proposed to act as the intermembrane space receptor for proteins [66]. Whilst detailed structural and mechanistic analysis has been carried out on this system in yeast [67], little is known about the components in other organisms. Interestingly the apparent lack of a gene encoding Mia40 in trypanosomes suggests that this pathway may display variations between species [68].

For the MIA machinery, orthologues of Mia40 and Erv1 are present in yeast, humans and plants (Figure 5A). Although Hot13 has been reported to be widespread in eukaryotes, analysis of the proteins identified indicates it is ~600 amino acids long and most likely a transcription factor in green plants. Brown algae contain a protein similar to yeast Hot13 (Figure 5A, Additional file 1). However, given the small size of this protein with conserved metal domains it is unclear if it is orthologous to the yeast protein. Although plant Erv1 and Mia40 are orthologous to their yeast counterparts, the primary structure of the protein differs (Figure 5B), suggesting possible mechanistic differences. Deletion of Mia40 in Arabidopsis is not lethal, and in fact normal growth and development are observed [69]. Erv1 is essential in Arabidopsis and analysis of the primary sequence indicates that the arrangement of cysteines differs to that in yeast (and humans). Arabidopsis Erv1 is similar to that found in *Trypanosoma brucei *and a protein called Erv2 that is located in the endoplasmic reticulum of yeast (which operates without a Mia40 like protein) (Figure 5B). Given that the import of the small Tim proteins and carrier proteins is normal in Arabidopsis plants that lack Mia40 [69], mechanistically Erv1 can function without Mia40 in oxidative protein folding in the intermembrane space. Analysis of the genome of *D. discoideum *indicates that Mia40 is present [68]. As Mia40 is absent in *Trypanosoma brucei*, *Encephalitozoon cuniculi *[65,68], and the brown algae *E. siliculosus *(Figure 5A, Additional file 1), this suggests that the presence of Mia40 in plants is a primitive situation, but its function(s) differ in various lineages.

### Translocases of the inner membrane (TIMS)

The inner mitochondrial membrane contains two translocases, the TIM17:23 complex that is responsible for the import of proteins via the general import pathway, and the TIM22 complex, that is responsible for the import of carrier proteins into the inner membrane. The TIM17:23 complex is responsible for the import of proteins that contain N-terminal targeting signals into or across the inner mitochondrial membrane [23]. This complex contains 9 components in yeast and several of the components are essential in yeast [52]. Tim23 forms a presequence and voltage sensitive channel [70], while Tim17 plays a crucial role in voltage sensing [71,72]. The TIM17:23 complex can be divided into the PAM complex, the presequence assisted motor consisting of five subunits (Tim44, HSP70, Pam 16, 17 and 18) and the membrane components of Tim17, 23, 21 and 50. The TIM22 translocase is responsible for the import of proteins that contain internal targeting signals and contain multiple (4 or 6) transmembrane spanning regions into the inner membrane [23]. In contrast to the TIM17:23 complex, the mechanistic details of how it operates are not yet fully understood. However Tim22 has been shown to have channel activity that is only active in the presence of a substrate protein [73]. In yeast it contains three accessory proteins, Tim54, 18 and 12 [52]. However no details on the composition of this complex in other organisms have been reported.

**Figure 6:**
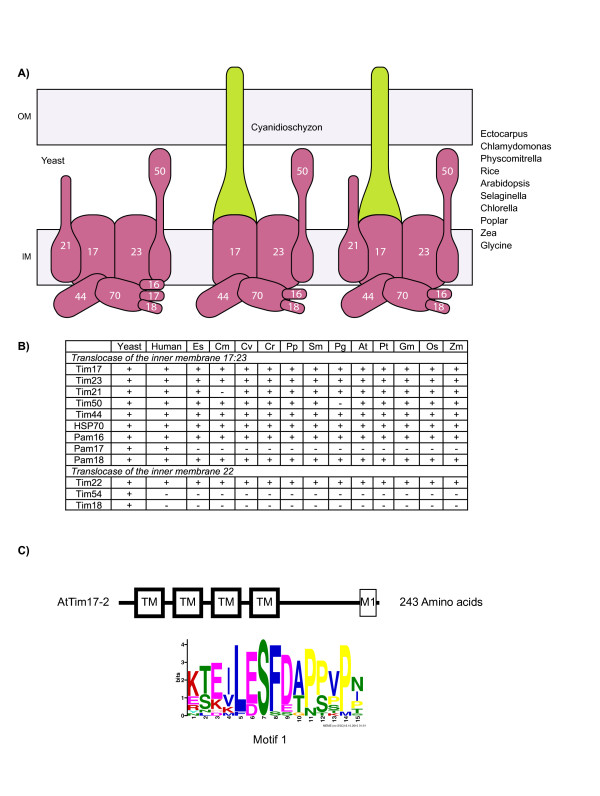
**The TIM17:23 and TIM22 complexes of plants**. A) A schematic representation of the TIM17:23 complex from yeast and plants. B) A table displaying the components of the TIM17:23 and TIM22 complexes in all plant species analysed. C) A diagram showing Arabidopsis Tim17-2 that contains four transmembrane domains (TM) and an extra C-terminal extension. This C-terminal extension is found in all higher plant species and motif analysis of the C-terminal extension identified to highly conserved regions of which both are related to nucleic acid binding proteins. Colors and abbreviations are the same as Figures 2 and 4.

In contrast to the TOM complex on the outer membrane, eight of the nine components of the Tim17:23 complex are conserved between yeast, humans and plants (the only difference being that yeast contain a Pam17 protein not present in humans and plants) (Figure 6A, Additional file 1). It has been previously proposed that the channel forming subunits of this complex, Tim23 and Tim17, are derived from amino acid transporters in bacteria, specifically LivH, and defined a family of proteins termed PReprotein and Amino acid Transporters (PRAT) [74]. It seems that originally there was one PRAT type protein that subsequently diverged to give rise to the three different PRAT proteins typically found in mitochondria [75]. In some organisms a single PRAT protein exists, which is likely a derived condition where the other PRAT proteins have been lost [65]. Additionally, not all subunits of the TIM17:23 complex are observed in all organisms, i.e. the absence of Tim50 in *D. discoideum *[49], suggests that accessory subunits can be lost.

In the case of the TIM22 complex, the translocase responsible for the import of metabolite carriers, or multiple spanning proteins of the inner membrane, including the PRAT proteins themselves, only the Tim22 component is conserved. In fact Tim18 and Tim54, along with Tim12, are only found in yeast and not in other organisms including plants. Thus, the additional components of this translocase are yet to be characterized in other organisms.

Although the TIMs seem to be better conserved in terms of orthology compared to the TOM complex, there are notable differences in plants. Firstly, the family of PRAT proteins has greatly expanded in plants compared to yeast and mammals. In Arabidopsis there are 17 members, rice has greater than 24 members and examination of *C. reinhardtii *and *P. patens *reveal 5 and 21 members respectively. Some of these PRAT proteins are located in plastids, while others are found in mitochondria [76]. In addition to the greater number of PRAT proteins in plant genomes, Tim17 in plants varies in size from 133 amino acids to 252 amino acids, the difference to yeast Tim17 of 158 amino acids is at the C-terminal end of the protein (Additional file 1). A C-terminal extension is found on Tim17 proteins from *C. merolae *through to Arabidopsis. It has been shown in Arabidopsis that this C-terminal extension is exposed on the outer surface of the outer membrane [36] and Arabidopsis Tim17 can only complement a yeast Tim17 mutant if this extension is removed [36].

In order to investigate possible function(s) of the Tim17 C-terminal extension in plants we conducted a motif search on all the identified Tim17 extensions. A distinct motif was detected (Figure 6C). Using this motif in blast searches identifies a number of different nucleic acid binding proteins (Additional file 2). This suggests that Tim17 in plants may be able to bind RNA and/or DNA. As plant mitochondria import tRNAs and have recently been shown to bind mRNA [22,77], suggests a possible role for Tim17 in the binding and/or import of nucleic acids into plant mitochondria.

### Mitochondrial processing peptidase(s)

Mitochondria require a number of peptidases to remove the targeting signals from proteins before or after they are assembled into functional protein complexes. These peptidases range from activities that remove the targeting signals, such as mitochondrial processing peptidase (MPP) and intermediate processing peptidase (IMP) [23], to the removal of a single amino acid from proteins that have already had the targeting signal removed [78], to the presequence degrading peptidase (PreP) that degrades the targeting signals once they have been removed [34].

Plant mitochondria contain a number of orthologous proteins in comparison with the various processing peptidases of yeast mitochondria (Additional file 1). At a sequence level the processing peptidases found in plant mitochondria look similar to those of yeast, however, functional investigations have revealed a number of differences. One major difference between yeast and plants is the location of the mitochondrial processing peptidase (MPP). In yeast both α and β MPP subunits are located in the matrix, however, in plants it has been demonstrated that they are integrated into the cytochrome bc_1 _complex located in the inner membrane [31,32]. Additionally, the presequence degrading peptidase of yeast is located in the intermembrane space whereas in plants it is located in the matrix [79,80]. Interestingly, the presequence degrading peptidase of plant mitochondria is also dual targeted to plastids where it degrades plastid targeting signals [79].

In terms of the processing site recognition by MPP in plants, it has been reported that the majority of plant mitochondrial presequences fall into 2 classes. In class 1 the processing signal is a -2 Arg residue while in class 2 presequences the signal is a -3 Arg residue [20,81], similar to what has been reported for yeast and mammals [23]. However, it has been demonstrated that in fact there is a second processing step in yeast [82], a novel peptidase called intermediate cleavage peptidase (Icp55) was found to process mitochondrial presequences after MPP, cleaving only 1 amino acid from the N-terminus, turning the proposed -2 cleavage signal into a -3 cleavage signal [78]. It is tempting to speculate that as plants contain an orthologue of Icp55 that the same cleavage is occurring, however this awaits experimental confirmation. Despite the orthology between many plant peptidases and those in other organisms it is still necessary to define their specific functions in plants. It has been demonstrated that the plant orthologue of the rhomboid protease from yeast does not carry out the same processing roles/activities in plants such as Arabidopsis [83].

## Conclusions

The plant mitochondrial import apparatus displays many differences compared to other non-plant organisms and between plant groups. The TOM complex in plants displays the most variability in that as many as five different TOM complexes exist in plants when red algae, brown algae and green plants are considered. Even in the green plant lineage variation is observed with OM64 only being present in monocot and dicot plants. While the composition of the other protein complexes may appear more conserved, the lack of biochemical characterization of these complexes in any plant group means that the presence of plant specific accessory subunits in various lineages cannot be judged. Additionally for some proteins, such as Mia40 and Tim17, functions have expanded in plants compared to those characterized in yeast.

## Methods

The protein sequences for all of the known mitochondrial protein import components from *Saccharomyces cerevisiae *(Tom20, Tom70, Tom71, Tom40, Tom22, Tom5, Tom6, Tom7, Sam50, Sam37, Sam35, Mdm10, Mim1, Mia40, Erv1, Hot13, Tim9, Tim10, Tim8, Tim13, Tim12, Tim22, Tim54, Tim18, Tim23, Tim17, Tim50, Tim21, mtHsp70, Mge1, Tim44, Pam18, Mdj2, Pam16, Pam17, MPPα, MPPβ, Oct1, Imp1, Imp2, Som1, Yta12, Yta10, Yme1, Mgr1, Mgr3, Pcp1, Icp55, Oxa1, Mba1, Cox18, Pnt1, Mss2, Mdj1, Hsp60, Hsp10, Hsp78 and Zim17) were downloaded from the NCBI protein database (http://www.ncbi.nlm.nih.gov/protein/). The Metaxin protein sequences were also obtained from *Homo sapiens*. Using the above protein sequences Blastp [84] searches of the protein sequences from *Physcomitrella patens*, *Selaginella moellendorffii, Chlamydomonas reinhardtii*, *Arabidopsis thaliana*, *Oryza sativa*, *Zea mays*, *Vitis vinifera*, *Glycine max *and *Populus tricocarpa *were performed using the Phytozome (http://www.phytozome.net) database. Blastp [84] searches of *Cyanidioschyzon merolae *were performed using the *Cyanidioschyzon merolae *genome project website (http://merolae.biol.s.u-tokyo.ac.jp/). Blastp [84] searches of *Ectocarpus siliculosus *were performed at the Bioinformatics online genome annotation system website (http://bioinformatics.psb.ugent.be/webtools/bogas/overview/Ectsi). Blastp [84] searches of *Chlorella variabilis NC64A *genome [38] was performed at the Chlorella genome website (http://genome.jgi-psf.org/ChlNC64A_1/ChlNC64A_1.home.html). To identify mitochondrial import components *of Picea glauca *tblastn [84] searches were carried on EST sequences [43] at the NCBI website (http://blast.ncbi.nlm.nih.gov/Blast.cgi).

All multiple sequence alignments were carried out using MAFFT [85] and visualized using Multiple align show (http://www.bioinformatics.org/sms/multi_align.html). The program IQPNNI [86] was used to reconstruct a maximum likelihood phylogeny assuming the Whelan and Goldman model [87]. Phylogenetic trees were finally visualized using the program Geneious (http://www.geneious.com).

TMpred (http://www.ch.embnet.org/software/TMPRED_form.html), TMHMM (http://www.cbs.dtu.dk/services/TMHMM/), and DAS (http://www.sbc.su.se/~miklos/DAS/) [88] were used in the prediction of transmembrane regions. TPR repeats were predicted using TPRpred (http://toolkit.tuebingen.mpg.de/tprpred) [89]. Motif analysis was performed using MEME (http://meme.nbcr.net/meme4_4_0/cgi-bin/meme.cgi) using default parameters for all plant like Metaxin sequences and sequences of the plant Tim17 extensions [90].

## Abbreviations

Erv1: Essential for respiration and vegetative growth 1; FAD: Flavin adenine dinucleotide; GDP: Guanosine diphosphate; GTP: Guanosine diphosphate; GST: Glutathione *S*-transferase; Hot13: Helper of Tim protein 13; Icp55: Intermediate cleavage peptidase of 55 kDa; Mdm10: Mitochondria distribution and morphology protein 10; MIA: Mitochondrial import and assembly; Mim1: Mitochondrial import 1; MPP: Mitochondrial processing peptidase; OM64: Mitochondrial outer membrane protein of 64 kDa; Omp85: Outer membrane protein of 85 kDa; PRAT: Preprotein and amino acid transporter; TIM: Translocase of the inner membrane; TOC: Translocase of the outer envelope of chloroplasts; TOM: Translocase of the outer membrane; TPR: Tetratricopeptide repeat; SAM: Sorting and assembly machinery

## Authors' contributions

CC carried out the data analysis with the help of MM. JW oversaw the analysis, design and implementation. CC, MM and JW drafted the manuscript. All authors read and approved final manuscript.

## Supplementary Material

Additional file 1 **Supplementary table 1. The mitochondrial import machinery of plants**. Click here for file

Additional file 2 **Supplementary table 2. The top 50 proteins identified using the conserved motifs on the C-terminal of plant Tim17 in a Blastp search**. Click here for file

## References

[B1] Cavalier-Smith T Predation and eukaryote cell origins: a coevolutionary perspective Int J Biochem Cell Biol 2009 41 2 307 322 10.1016/j.biocel.2008.10.002 18935970

[B2] Dyall SD Brown MT Johnson PJ Ancient invasions: from endosymbionts to organelles Science 2004 304 5668 253 257 10.1126/science.1094884 15073369

[B3] Mentel M Martin W Energy metabolism among eukaryotic anaerobes in light of Proterozoic ocean chemistry Philos Trans R Soc Lond B Biol Sci 2008 363 1504 2717 2729 10.1098/rstb.2008.0031 18468979 PMC2606767

[B4] Deusch O Landan G Roettger M Gruenheit N Kowallik KV Allen JF Martin W Dagan T Genes of cyanobacterial origin in plant nuclear genomes point to a heterocyst-forming plastid ancestor Mol Biol Evol 2008 25 4 748 761 10.1093/molbev/msn022 18222943

[B5] Gould SB Waller RF McFadden GI Plastid evolution Annu Rev Plant Biol 2008 59 491 517 10.1146/annurev.arplant.59.032607.092915 18315522

[B6] Theissen U Martin W The difference between organelles and endosymbionts Curr Biol 2006 16 24 R1016 1017 author reply R1017-1018 10.1016/j.cub.2006.11.020 17174902

[B7] Huang CY Ayliffe MA Timmis JN Direct measurement of the transfer rate of chloroplast DNA into the nucleus Nature 2003 422 6927 72 76 10.1038/nature01435 12594458

[B8] Adams KL Palmer JD Evolution of mitochondrial gene content: gene loss and transfer to the nucleus Mol Phylogenet Evol 2003 29 3 380 395 10.1016/S1055-7903(03)00194-5 14615181

[B9] Martin W Herrmann RG Gene transfer from organelles to the nucleus: how much, what happens, and Why? Plant Physiol 1998 118 1 9 17 10.1104/pp.118.1.9 9733521 PMC1539188

[B10] Lithgow T Schneider A Evolution of macromolecular import pathways in mitochondria, hydrogenosomes and mitosomes Philos Trans R Soc Lond B Biol Sci 2010 365 1541 799 817 10.1098/rstb.2009.0167 20124346 PMC2817224

[B11] Alverson AJ Wei X Rice DW Stern DB Barry K Palmer JD Insights into the evolution of mitochondrial genome size from complete sequences of Citrullus lanatus and Cucurbita pepo (Cucurbitaceae) Mol Biol Evol 2010 20118192 10.1093/molbev/msq029 PMC2877997

[B12] Bonen L Cis- and trans-splicing of group II introns in plant mitochondria Mitochondrion 2008 8 1 26 34 10.1016/j.mito.2007.09.005 18006386

[B13] Palmer JD Herbon LA Plant mitochondrial DNA evolves rapidly in structure, but slowly in sequence J Mol Evol 1989 28 87 97 10.1007/BF02143500 3148746

[B14] Wolfe KH Li WH Sharp PM Rates of nucleotide substitution vary greatly among plant mitochondrial, chloroplast, and nuclear DNAs Proc Natl Acad Sci USA 1987 84 24 9054 9058 10.1073/pnas.84.24.9054 3480529 PMC299690

[B15] Schmitz-Linneweber C Small I Pentatricopeptide repeat proteins: a socket set for organelle gene expression Trends Plant Sci 2008 13 12 663 670 10.1016/j.tplants.2008.10.001 19004664

[B16] Richardson AO Palmer JD Horizontal gene transfer in plants J Exp Bot 2007 58 1 1 9 10.1093/jxb/erl148 17030541

[B17] Day DA Whelna J Millar AH Siedow J Wiskich JT REgulation of the alternative oxidase in Plants and Fungi Aus J Plant Physiol 1995 22 497 509 10.1071/PP9950497

[B18] Considine MJ Holtzapffel RC Day DA Whelan J Millar AH Molecular distinction between alternative oxidase from monocots and dicots Plant Physiol 2002 129 3 949 953 10.1104/pp.004150 12114550 PMC1540239

[B19] Somerville C Browse J Ohlrogge J Buchanan BB, Gruissem W, Jones RL Lipids Biochemistry and Molecular Biology of Plants 2002 Wiley 456 527

[B20] Zhang XP Glaser E Interaction of plant mitochondrial and chloroplast signal peptides with the Hsp70 molecular chaperone Trends Plant Sci 2002 7 1 14 21 10.1016/S1360-1385(01)02180-X 11804822

[B21] Chew O Whelan J Just read the message: a model for sorting of proteins between mitochondria and chloroplasts Trends Plant Sci 2004 9 7 318 319 10.1016/j.tplants.2004.05.003 15231275

[B22] Michaud M Marechal-Drouard L Duchene AM RNA trafficking in plant cells: targeting of cytosolic mRNAs to the mitochondrial surface Plant Mol Biol 20506035 10.1007/s11103-010-9650-3

[B23] Neupert W Herrmann JM Translocation of proteins into mitochondria Annu Rev Biochem 2007 76 723 749 10.1146/annurev.biochem.76.052705.163409 17263664

[B24] Soll J Schleiff E Protein import into chloroplasts Nat Rev Mol Cell Biol 2004 5 3 198 208 10.1038/nrm1333 14991000

[B25] Jansch L Kruft V Schmitz UK Braun HP Unique composition of the preprotein translocase of the outer mitochondrial membrane from plants J Biol Chem 1998 273 27 17251 17257 10.1074/jbc.273.27.17251 9642296

[B26] Werhahn W Niemeyer A Jänsch L Kruft V Schmitz UK Braun HP Purification and characterization of the preprotein translocase of the outer mitochondrial membrane from Arabidopsis. Identification of multiple forms of TOM20 Plant Physiology 2001 125 943 954 10.1104/pp.125.2.943 11161051 PMC64895

[B27] Perry AJ Hulett JM Likic VA Lithgow T Gooley PR Convergent evolution of receptors for protein import into mitochondria Curr Biol 2006 16 3 221 229 10.1016/j.cub.2005.12.034 16461275

[B28] Lister R MW M Whelan J The Mitochondrial Protein Import Machinery of Plants (MPIMP) database Nucleic Acids Reserach 2003 31 325 327 10.1093/nar/gkg055 PMC165502 12520014

[B29] Chan NC Likic VA Waller RF Mulhern TD Lithgow T The C-terminal TPR domain of Tom70 defines a family of mitochondrial protein import receptors found only in animals and fungi J Mol Biol 2006 358 4 1010 1022 10.1016/j.jmb.2006.02.062 16566938

[B30] Macasev D Whelan J Newbigin E Silva-Filho MC Mulhern TD Lithgow T Tom22', an 8-kDa trans-site receptor in plants and protozoans, is a conserved feature of the TOM complex that appeared early in the evolution of eukaryotes Mol Biol Evol 2004 21 8 1557 1564 10.1093/molbev/msh166 15155803

[B31] Braun HP Emmermann M Kruft V Schmitz UK The general mitochondrial processing peptidase from potato is an integral part of cytochrome c reductase of the respiratory chain EMBO J 1992 11 9 3219 3227 1324169 10.1002/j.1460-2075.1992.tb05399.x PMC556855

[B32] Glaser E Dessi P Integration of the mitochondrial-processing peptidase into the cytochrome bc1 complex in plants J Bioenerg Biomembr 1999 31 3 259 274 10.1023/A:1005475930477 10591532

[B33] Brumme S Kruft V Schmitz UK Braun HP New insights into the co-evolution of cytochrome c reductase and the mitochondrial processing peptidase J Biol Chem 1998 273 13143 13149 10.1074/jbc.273.21.13143 9582354

[B34] Bhushan S Ståhl A Nilsson S Lefebvre B Seki M Roth C McWilliam D Wright SJ Liberles DA Shinozaki K Catalysis, subcellular localization, expression and evolution of the targeting peptides degrading protease, AtPreP2 Plant Cell Physiology 2005 46 985 996 10.1093/pcp/pci107 15827031

[B35] Lister R Mowday B Whelan J AH M Zinc-dependent intermembrane space proteins stimulate import of carrier proteins into plant mitochondria Plant J 2002 30 555 566 10.1046/j.1365-313X.2002.01316.x 12047630

[B36] Murcha MW Elhafez D Millar AH Whelan J The C-terminal region of TIM17 links the outer and inner mitochondrial membranes in Arabidopsis and is essential for protein import J Biol Chem 2005 280 16 16476 16483 10.1074/jbc.M413299200 15722347

[B37] Merchant SS Prochnik SE Vallon O Harris EH Karpowicz SJ Witman GB Terry A Salamov A Fritz-Laylin LK Marechal-Drouard L The Chlamydomonas genome reveals the evolution of key animal and plant functions Science 2007 318 5848 245 250 10.1126/science.1143609 17932292 PMC2875087

[B38] Blanc G Duncan G Agarkova I Borodovsky M Gurnon J Kuo A Lindquist E Lucas S Pangilinan J Polle J The Chlorella variabilis NC64A Genome Reveals Adaptation to Photosymbiosis, Coevolution with Viruses, and Cryptic Sex Plant Cell10.1105/tpc.110.076406 PMC2965543 20852019

[B39] Rensing SA Lang D Zimmer AD Terry A Salamov A Shapiro H Nishiyama T Perroud PF Lindquist EA Kamisugi Y The Physcomitrella genome reveals evolutionary insights into the conquest of land by plants Science 2008 319 5859 64 69 10.1126/science.1150646 18079367

[B40] Banks JA Selaginella and 400 million years of separation Annu Rev Plant Biol 2009 60 223 238 10.1146/annurev.arplant.59.032607.092851 19575581

[B41] The map-based sequence of the rice genome Nature 2005 436 7052 793 800 10.1038/nature03895 16100779

[B42] Arabidopsis Genome Initiative Nature 2000 408 6814 796 815 10.1038/35048692 11130711

[B43] Pavy N Paule C Parsons L Crow JA Morency MJ Cooke J Johnson JE Noumen E Guillet-Claude C Butterfield Y Generation, annotation, analysis and database integration of 16,500 white spruce EST clusters BMC Genomics 2005 6 144 10.1186/1471-2164-6-144 16236172 PMC1277824

[B44] Cock JM Sterck L Rouze P Scornet D Allen AE Amoutzias G Anthouard V Artiguenave F Aury JM Badger JH The Ectocarpus genome and the independent evolution of multicellularity in brown algae Nature 465 7298 617 621 10.1038/nature09016 20520714

[B45] Matsuzaki M Misumi O Shin IT Maruyama S Takahara M Miyagishima SY Mori T Nishida K Yagisawa F Yoshida Y Genome sequence of the ultrasmall unicellular red alga Cyanidioschyzon merolae 10D Nature 2004 428 6983 653 657 10.1038/nature02398 15071595

[B46] Keeling PJ The endosymbiotic origin, diversification and fate of plastids Philos Trans R Soc Lond B Biol Sci 2010 365 1541 729 748 10.1098/rstb.2009.0103 20124341 PMC2817223

[B47] Bolte K Bullmann L Hempel F Bozarth A Zauner S Maier UG Protein targeting into secondary plastids J Eukaryot Microbiol 2009 56 1 9 15 10.1111/j.1550-7408.2008.00370.x 19335770

[B48] Dimmer KS Rapaport D Proteomic view of mitochondrial function Genome Biol 2008 9 2 209 10.1186/gb-2008-9-2-209 18331620 PMC2374722

[B49] Dolezal P Dagley MJ Kono M Wolynec P Likic VA Foo JH Sedinova M Tachezy J Bachmann A Bruchhaus I The essentials of protein import in the degenerate mitochondrion of Entamoeba histolytica PLoS Pathog 2010 6 3 e1000812 10.1371/journal.ppat.1000812 20333239 PMC2841616

[B50] Likic VA Dolezal P Celik N Dagley M Lithgow T Using hidden markov models to discover new protein transport machines Methods Mol Biol 2010 619 271 284 full_text 20419416 10.1007/978-1-60327-412-8_16

[B51] van Wilpe S Ryan MT Hill K Maarse AC Meisinger C Brix J Dekker PJ Moczko M Wagner R Meijer M Tom22 is a multifunctional organizer of the mitochondrial preprotein translocase Nature 1999 401 6752 485 489 10.1038/46802 10519552

[B52] Chacinska A Koehler CM Milenkovic D Lithgow T Pfanner N Importing mitochondrial proteins: machineries and mechanisms Cell 2009 138 4 628 644 10.1016/j.cell.2009.08.005 19703392 PMC4099469

[B53] Lister R Carrie C Duncan O Ho LH Howell KA Murcha MW Whelan J Functional definition of outer membrane proteins involved in preprotein import into mitochondria Plant Cell 2007 19 11 3739 3759 10.1105/tpc.107.050534 17981999 PMC2174869

[B54] Likic VA Perry A Hulett J Derby M Traven A Waller RF Keeling PJ Koehler CM Curran SP Gooley PR Patterns that define the four domains conserved in known and novel isoforms of the protein import receptor Tom20 J Mol Biol 2005 347 1 81 93 10.1016/j.jmb.2004.12.057 15733919

[B55] Lister R Whelan J Mitochondrial protein import: convergent solutions for receptor structure Curr Biol 2006 16 6 R197 199 10.1016/j.cub.2006.02.024 16546069

[B56] Chew O Lister R Qbadou S Heazlewood JL Soll J Schleiff E Millar AH Whelan J A plant outer mitochondrial membrane protein with high amino acid sequence identity to a chloroplast protein import receptor FEBS Lett 2004 557 1-3 109 114 10.1016/S0014-5793(03)01457-1 14741350

[B57] Yoon HS Hackett JD Ciniglia C Pinto G Bhattacharya D A molecular timeline for the origin of photosynthetic eukaryotes Mol Biol Evol 2004 21 5 809 818 10.1093/molbev/msh075 14963099

[B58] Patron NJ Waller RF Transit peptide diversity and divergence: A global analysis of plastid targeting signals Bioessays 2007 29 10 1048 1058 10.1002/bies.20638 17876808

[B59] Gentle IE Burri L Lithgow T Molecular architecture and function of the Omp85 family of proteins Mol Microbiol 2005 58 5 1216 1225 10.1111/j.1365-2958.2005.04906.x 16313611

[B60] Paschen SA Waizenegger T Stan T Preuss M Cyrklaff M Hell K Rapaport D Neupert W Evolutionary conservation of biogenesis of beta-barrel membrane proteins Nature 2003 426 6968 862 866 10.1038/nature02208 14685243

[B61] Kozjak-Pavlovic V Ross K Benlasfer N Kimmig S Karlas A Rudel T Conserved roles of Sam50 and metaxins in VDAC biogenesis EMBO Rep 2007 8 6 576 582 10.1038/sj.embor.7400982 17510655 PMC2002532

[B62] Schneider A Bursac D Lithgow T The direct route: a simplified pathway for protein import into the mitochondrion of trypanosomes Trends Cell Biol 2008 18 1 12 18 10.1016/j.tcb.2007.09.009 18068984

[B63] Dolezal P Likic V Tachezy J Lithgow T Evolution of the molecular machines for protein import into mitochondria Science 2006 313 5785 314 318 10.1126/science.1127895 16857931

[B64] Gentle IE Perry AJ Alcock FH Likic VA Dolezal P Ng ET Purcell AW McConnville M Naderer T Chanez AL Conserved motifs reveal details of ancestry and structure in the small TIM chaperones of the mitochondrial intermembrane space Mol Biol Evol 2007 24 5 1149 1160 10.1093/molbev/msm031 17329230

[B65] Waller RF Jabbour C Chan NC Celik N Likic VA Mulhern TD Lithgow T Evidence of a reduced and modified mitochondrial protein import apparatus in microsporidian mitosomes Eukaryot Cell 2009 8 1 19 26 10.1128/EC.00313-08 19028997 PMC2620743

[B66] Riemer J Fischer M Herrmann JM Oxidation-driven protein import into mitochondria: Insights and blind spots Biochim Biophys Acta 2010 20537978 10.1016/j.bbamem.2010.06.003

[B67] Endo T Yamano K Kawano S Structural basis for the disulfide relay system in the mitochondrial intermembrane space Antioxid Redox Signal 2010 13 9 1359 1373 10.1089/ars.2010.3099 20136511

[B68] Allen JW Ferguson SJ Ginger ML Distinctive biochemistry in the trypanosome mitochondrial intermembrane space suggests a model for stepwise evolution of the MIA pathway for import of cysteine-rich proteins FEBS Lett 2008 582 19 2817 2825 10.1016/j.febslet.2008.07.015 18639549

[B69] Carrie C Giraud E Duncan O Xu H Wang Y Clifton R Murcha MW Filipovska A Rackham O Vrielink A Characterisation of the disulfide relay system of Arabidopsis thaliana mitochondria reveals that Erv1 is the only known essential component 2010 in press

[B70] Truscott KN Kovermann P Geissler A Merlin A Meijer M Driessen AJ Rassow J Pfanner N Wagner R A presequence- and voltage-sensitive channel of the mitochondrial preprotein translocase formed by Tim23 Nat Struct Biol 2001 8 12 1074 1082 10.1038/nsb726 11713477

[B71] Martinez-Caballero S Grigoriev SM Herrmann JM Campo ML Kinnally KW Tim17p regulates the twin pore structure and voltage gating of the mitochondrial protein import complex TIM23 J Biol Chem 2007 282 6 3584 3593 10.1074/jbc.M607551200 17148445

[B72] Meier S Neupert W Herrmann JM Conserved N-terminal negative charges in the Tim17 subunit of the TIM23 translocase play a critical role in the import of preproteins into mitochondria J Biol Chem 2005 280 9 7777 7785 10.1074/jbc.M412158200 15618217

[B73] Peixoto PM Grana F Roy TJ Dunn CD Flores M Jensen RE Campo ML Awaking TIM22, a dynamic ligand-gated channel for protein insertion in the mitochondrial inner membrane J Biol Chem 2007 282 26 18694 18701 10.1074/jbc.M700775200 17462993

[B74] Rassow J Dekker PJ van Wilpe S Meijer M Soll J The preprotein translocase of the mitochondrial inner membrane: function and evolution J Mol Biol 1999 286 1 105 120 10.1006/jmbi.1998.2455 9931253

[B75] Clements A Bursac D Gatsos X Perry AJ Civciristov S Celik N Likic VA Poggio S Jacobs-Wagner C Strugnell RA The reducible complexity of a mitochondrial molecular machine Proc Natl Acad Sci USA 2009 106 37 15791 15795 10.1073/pnas.0908264106 19717453 PMC2747197

[B76] Murcha MW Elhafez D Lister R Tonti-Filippini J Baumgartner M Philippar K Carrie C Mokranjac D Soll J Whelan J Characterization of the preprotein and amino acid transporter gene family in Arabidopsis Plant Physiol 2007 143 1 199 212 10.1104/pp.106.090688 17098851 PMC1761978

[B77] Eliyahu E Pnueli L Melamed D Scherrer T Gerber AP Pines O Rapaport D Arava Y Tom20 mediates localization of mRNAs to mitochondria in a translation-dependent manner Mol Cell Biol 30 1 284 294 10.1128/MCB.00651-09 19858288 PMC2798288

[B78] Vogtle FN Wortelkamp S Zahedi RP Becker D Leidhold C Gevaert K Kellermann J Voos W Sickmann A Pfanner N Global analysis of the mitochondrial N-proteome identifies a processing peptidase critical for protein stability Cell 2009 139 2 428 439 10.1016/j.cell.2009.07.045 19837041

[B79] Bhushan S Lefebvre B Stahl A Wright SJ Bruce BD Boutry M Glaser E Dual targeting and function of a protease in mitochondria and chloroplasts EMBO Rep 2003 4 11 1073 1078 10.1038/sj.embor.7400011 14578924 PMC1326381

[B80] Kambacheld M Augustin S Tatsuta T Muller S Langer T Role of the novel metallopeptidase Mop112 and saccharolysin for the complete degradation of proteins residing in different subcompartments of mitochondria J Biol Chem 2005 280 20 20132 20139 10.1074/jbc.M500398200 15772085

[B81] Huang S Taylor NL Whelan J Millar AH Refining the definition of plant mitochondrial presequences through analysis of sorting signals, N-terminal modifications, and cleavage motifs Plant Physiol 2009 150 3 1272 1285 10.1104/pp.109.137885 19474214 PMC2705053

[B82] Schneider G Sjoling S Wallin E Wrede P Glaser E von Heijne G Feature-extraction from endopeptidase cleavage sites in mitochondrial targeting peptides Proteins 1998 30 1 49 60 10.1002/(SICI)1097-0134(19980101)30:1<49::AID-PROT5>3.0.CO;2-F 9443340

[B83] Kmiec-Wisniewska B Krumpe K Urantowka A Sakamoto W Pratje E Janska H Plant mitochondrial rhomboid, AtRBL12, has different substrate specificity from its yeast counterpart Plant Mol Biol 2008 68 1-2 159 171 10.1007/s11103-008-9359-8 18543065

[B84] Altschul SF Gish W Miller W Myers EW Lipman DJ Basic local alignment search tool J Mol Biol 1990 215 3 403 410 2231712 10.1016/S0022-2836(05)80360-2

[B85] Katoh K Kuma K Toh H Miyata T MAFFT version 5: improvement in accuracy of multiple sequence alignment Nucleic Acids Res 2005 33 2 511 518 10.1093/nar/gki198 15661851 PMC548345

[B86] Vinh le S Von Haeseler A IQPNNI: moving fast through tree space and stopping in time Mol Biol Evol 2004 21 8 1565 1571 10.1093/molbev/msh176 15163768

[B87] Whelan S Goldman N A general empirical model of protein evolution derived from multiple protein families using a maximum-likelihood approach Mol Biol Evol 2001 18 5 691 699 11319253 10.1093/oxfordjournals.molbev.a003851

[B88] Cserzo M Wallin E Simon I von Heijne G Elofsson A Prediction of transmembrane alpha-helices in prokaryotic membrane proteins: the dense alignment surface method Protein Eng 1997 10 6 673 676 10.1093/protein/10.6.673 9278280

[B89] Karpenahalli MR Lupas AN Soding J TPRpred: a tool for prediction of TPR-, PPR- and SEL1-like repeats from protein sequences BMC Bioinformatics 2007 8 2 10.1186/1471-2105-8-2 17199898 PMC1774580

[B90] Bailey TL Elkan C Fitting a mixture model by expectation maximization to discover motifs in biopolymers Proc Int Conf Intell Syst Mol Biol 1994 2 28 36 7584402

[B91] Fredslund J PHY.FI: fast and easy online creation and manipulation of phylogeny color figures BMC Bioinformatics 2006 7 315 10.1186/1471-2105-7-315 16792795 PMC1513607

